# Dissociations between spatial-attentional processes within parietal cortex: insights from hybrid spatial cueing and change detection paradigms

**DOI:** 10.3389/fnhum.2013.00366

**Published:** 2013-07-16

**Authors:** Rik Vandenberghe, Céline R. Gillebert

**Affiliations:** ^1^Laboratory for Cognitive Neurology, Department of Neurosciences, Katholieke Universiteit LeuvenLeuven, Belgium; ^2^Neurology Department, University Hospitals LeuvenLeuven, Belgium; ^3^Department of Experimental Psychology, University of OxfordOxford, UK

**Keywords:** area PF, temporoparietal junction, intraparietal sulcus, superior parietal lobule, invalidity, attentional priority map

## Abstract

Spatial cueing has been used by many different groups under multiple forms to study spatial attention processes. We will present evidence obtained in brain-damaged patients and healthy volunteers using a variant of this paradigm, the hybrid spatial cueing paradigm, which, besides single-target trials with valid and invalid cues, also contains trials where a target is accompanied by a contralateral competing stimulus (competition trials). This allows one to study invalidity-related processes and selection between competing stimuli within the same paradigm. In brain-damaged patients, lesions confined to the intraparietal sulcus result in contralesional attentional deficits, both during competition and invalid trials, according to a pattern that does not differ from that observed following inferior parietal lesions. In healthy volunteers, however, selection between competing stimuli and invalidity-related processes are partially dissociable, the former relying mainly on cytoarchitectonic areas hIP1-3 in the intraparietal sulcus, the latter on cytoarchitectonic area PF in the right inferior parietal lobule. The activity profile in more posterior inferior parietal areas PFm and PGa, does not distinguish between both types of trials. The functional account for right PF and adjacent areas is further constrained by the activity profile observed during other experimental paradigms. In a change detection task with variable target and distracter set size, for example, these inferior parietal areas show highest activity when the stimulus array consists of only one single target, while the intraparietal sulcus show increased activity as the array contains more targets and distracters. Together, these findings lead us to the hypothesis that right PF functions as a target singleton detector, which is activated when a target stands out from the background, referring both to the temporal background (expectancy) and the momentaneous background (stimulus-driven saliency).

## 1. Introduction

Spatial attention encompasses a wide set of divergent processes that govern the distribution of attentional weights over locations that are, or may be, occupied by objects. A powerful concept in spatial attention research, stemming from neurophysiology and computational neurobiology, is the “attentional priority map”. The attentional priority map refers to a topographic representation of attentional weights (Bushnell et al., 1981; Koch and Ullman, 1985; Colby et al., 1996; Gottlieb et al., 1998; Itti and Koch, 2001; Bisley and Goldberg, 2003; Vandenberghe and Gillebert, 2009; Ptak, 2012; Jerde and Curtis, 2013). The attentional weights depend, among other variables, on sensory evidence (Bundesen and Habekost, 2008) obtained through multiple input channels (visual, auditory, …). The current review will be restricted to effects obtained within the visual modality. Although attentional priorities may be sustained over a prolonged period of time (Vandenberghe et al., 2001a,b; Husain and Rorden, 2003), most evidence with regards to parietal cortex relates to its role in transitions between attentional priority maps (Vandenberghe et al., 2001a; Molenberghs et al., 2007). Here we will describe novel evidence obtained using two paradigms, the hybrid spatial cueing paradigm (Gillebert et al., 2011, 2012a, 2013) and the change detection paradigm with varying target and distracter set size (Gillebert et al., 2012b), in patients (Gillebert et al., 2011) and in the intact brain (Gillebert et al., 2012a,b, 2013), both from a localizationist and a connectionist perspective.

## 2. The hybrid spatial cueing task

### 2.1. Converging evidence from functional imaging and patient lesion data

**Figure 1:**
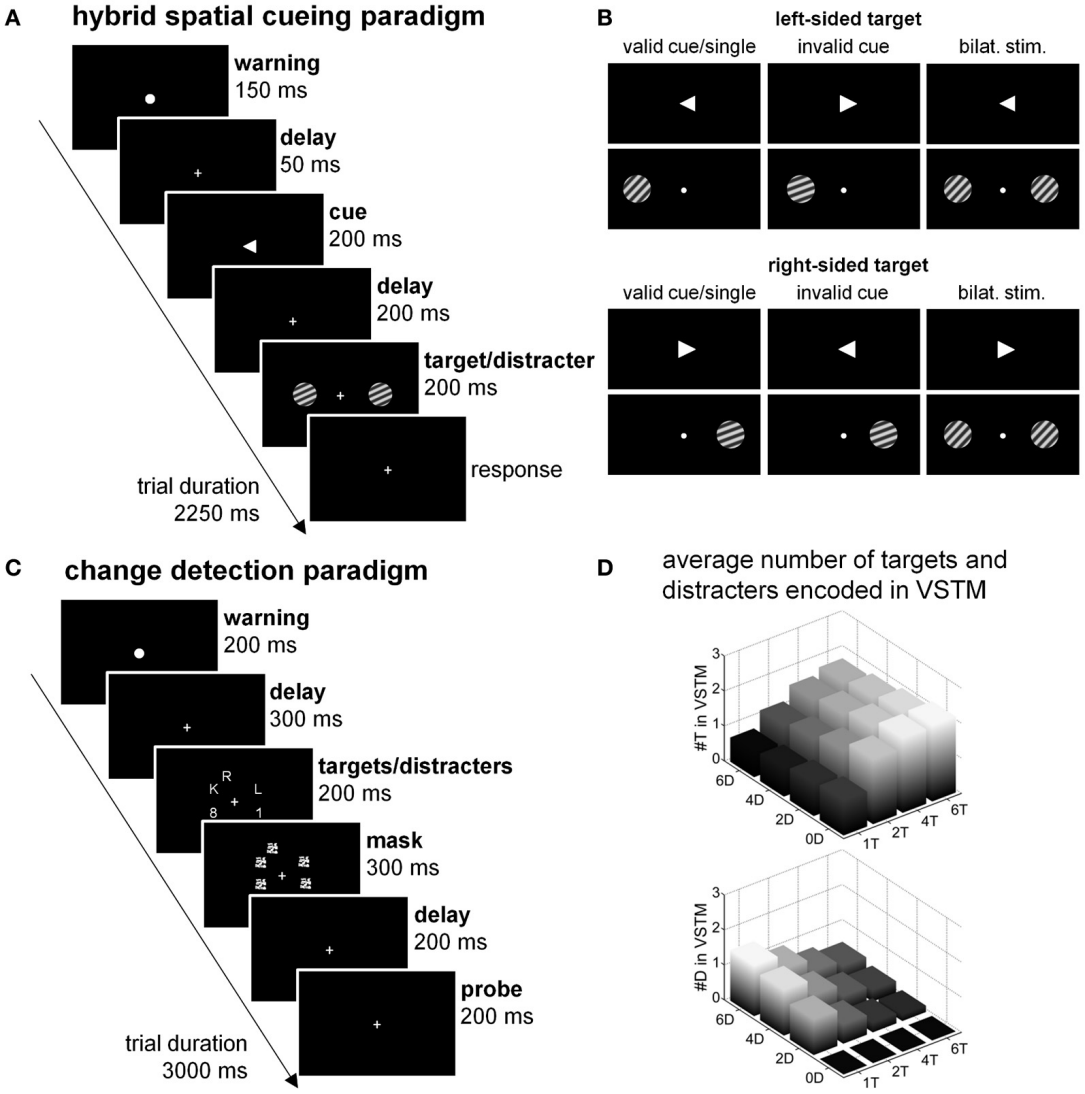
**Experimental paradigms at the focus of the current review. (A,B)** Hybrid spatial cueing paradigm. **(A)** Timing. **(B)** Spatial cueing with single validly cued target, invalidly cued trials (20%) and competition trials (20%). For the competition trials proper sensory control experiments were conducted to tease out attentional and sensory effects of adding a distracter. **(C,D)** Change detection paradigm. **(C)** Timing and basic design. In reality the target set size was varied in discrete steps (1, 2, 4, or 6 targets) as well as the distracter set size (0, 2, 4, or 6 distracters) according to a factorial design. Letters were targets, numbers distracters. **(D)** Factorial design with varying targets (T) and distracters (D) within the array. The height of the bars corresponds to the number of targets (#T in VSTM) and the number of distracters (#D in VSTM) loaded into visual short-term memory (VSTM) in each of the cells of the factorial design, according to the Theory of Visual Attention.

Numerous experiments in humans have provided converging evidence for the distinct role of different parietal regions in spatial attention (for reviews, see Vandenberghe and Gillebert, 2009; Vandenberghe et al., 2012). Here we will focus on the hybrid spatial cueing paradigm (Figures 1A,B) which allows one to simultaneously study two key operations of spatially selective attention: selection between competing stimuli (Desimone and Duncan, 1995) and the processing of invalidly cued targets (Corbetta et al., 2000). The hybrid spatial cueing paradigm enables one to contrast the neuroanatomy of both processes within the same subjects, both in healthy volunteers and in patients with parietal brain damage, and to relate the findings to the cytoarchitectonic organization of the parietal cortex, provided that proper sensory control conditions are used (Vandenberghe et al., 2005; Molenberghs et al., 2008; Gillebert et al., 2013). Compared to baseline, the validly cued single-grating trials require interpretation of the central arrow cue (Woldorff et al., 2004; Bonato et al., 2009), assignment of a high attentional weight to the cued peripheral location, short-term maintenance of that weight, detection of the grating at the cued location and discrimination of its orientation, response selection based on a conditional-associative rule and response execution. Compared to the validly cued single-grating trials, the presence of an irrelevant contralateral distracter induces a need to select a target among nearly identical distracting stimuli on the basis of the instructional spatial cue, and to suppress undue interference by the distracter's orientation on response selection. Behaviorally, competition trials are usually associated with a cost compared to valid single-grating trials (Vandenberghe et al., 2005; Molenberghs et al., 2008). By titrating the orientation difference to be discriminated, performance measures during functional magnetic resonance imaging (fMRI) can be strictly matched between the two trial types, removing the potential confound of aspecific differences in task difficulty (Vandenberghe et al., 2005; Molenberghs et al., 2008). The addition of a distracter also causes a sensory mismatch with the valid single-grating trials. Additional sensory control trials therefore are necessary to distinguish attentional effects from the sensory effect induced by adding a distracter (Vandenberghe et al., 2005; Molenberghs et al., 2008; Gillebert et al., 2013). The cognitive processes induced by invalidly cued trials have been intensively studied and described before [see Corbetta et al. (2008) for a review]. Briefly, with the delay durations and trial frequencies we used, when an invalidly cued grating appears, subjects have to detect the grating at the unexpected location and shift attention from the predicted location to the target location.

**Figure 2:**
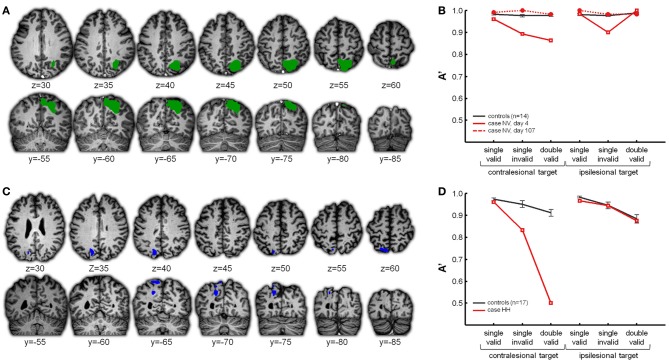
**(A,B)** Case NV. **(A)** In green the right middle IPS lesion in case NV, affecting the horizontal segment of IPS with extension into the superior parietal lobule. **(B)** NV's accuracy (expressed as A′) obtained in the different conditions of the hybrid spatial cueing paradigm (red), compared to age-matched controls (black). NV was tested on two instances. On day 4 (full red line) the lesion was as visualized in **(A)**, on day 107 the lesion had substantially regressed (dotted red line). For more details see Gillebert et al. (2011). **(C,D)** Case HH. **(C)** In blue the left posterior IPS lesion in case HH. **(D)** HH's performance obtained in the different conditions of the hybrid spatial cueing paradigm (red), compared to age-matched controls (black).

When subjects have to select between competing stimuli, the middle segment of the intraparietal sulcus (IPS) is consistently more active compared to single stimulus conditions (Vandenberghe et al., 2005; Molenberghs et al., 2008; Gillebert et al., 2012a, 2013) This difference in middle IPS activity between double stimulation and single grating stimulation is absent under sensory control conditions where attention is directed centrally (Vandenberghe et al., 2005; Molenberghs et al., 2008; Gillebert et al., 2013). When the functional activity map obtained in healthy volunteers by contrasting competition trials vs. single-grating trials is overlaid with the voxel-based lesion-symptom map obtained in patients with unifocal cortical stroke from a closely similar contrast, the overlap is situated at the lower bank of middle IPS (Molenberghs et al., 2008). This provides evidence that the deficit in spatially selective attention following inferior parietal lesions can be accounted for by extension of the lesions into the lower bank of the middle IPS which is also activated in healthy controls (Molenberghs et al., 2008). Further evidence for the critical role of middle IPS in this selective attention paradigm comes from a detailed study of a case with a reversible lesion confined to middle IPS with extension into the superior parietal lobule caused by a venous sinus thrombosis, case NV (Figure 2A): the lesion provoked a deficit during spatial cueing when a distracter was present ipsilesionally compared to single-stimulus conditions and also during invalidly cued trials (Gillebert et al., 2011) (Figure 2B). The effect of adding a distracter was limited to the contralesional target conditions while the effect on invalidly cued trials was present for both ipsi- and contralesional targets (Gillebert et al., 2011; Vandenberghe et al., 2012). The shifting deficit for both left- and rightward attention may possibly be due to the extension into the superior parietal lobule (see below) (Vandenberghe et al., 2001a; Yantis et al., 2002; Molenberghs et al., 2007). When the lesion partially regressed due to the resolution of vasogenic edema, the behavioral deficit also recovered (Figure 2B) (Gillebert et al., 2011).

**Figure 3:**
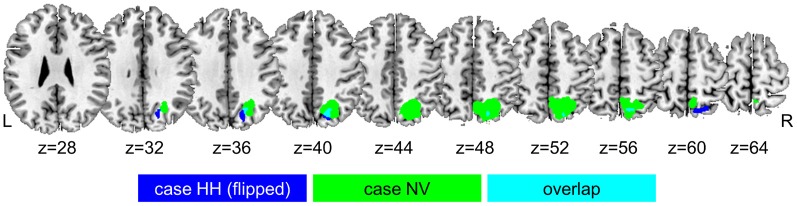
**Overlap between the right-sided middle IPS lesion in case NV (in green) and the left-sided posterior IPS lesion in case HH (in blue), after flipping the lesion of case HH from the left to the right hemisphere**.

**Figure 4:**
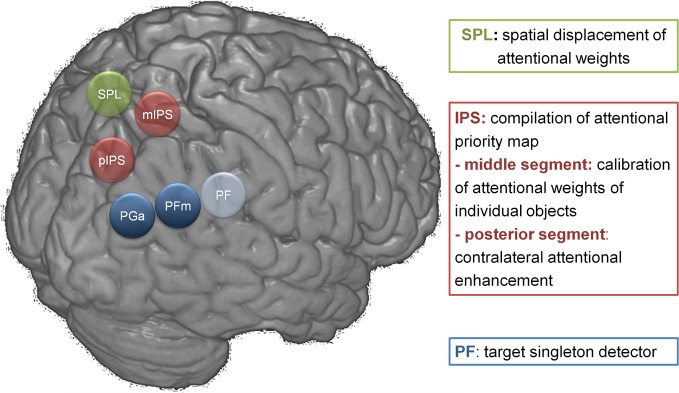
**Functional-anatomical model of parietal function**. PGa and PFm have not received a functional characterization in this overview figure. Their contribution to cognitive processing across a wide variety of domains is the focus of other recent reviews (Duncan, 2010; Seghier, 2013; Cabeza et al., 2012). The cytoarchitectonic labeling of inferior parietal cortex is based on Caspers et al. (2006). pIPS, posterior (descending) segment of IPS; mIPS, middle (horizontal) segment of IPS; SPL, superior parietal lobule.

Studies of rare focal lesions in parietal cortex have been fruitful in elucidating the critical role of specific parietal areas during spatially selective attention and reorienting. Such lesions can have a size of only one or a few cm^3^, sparing white matter tracts and sometimes with only limited effects on connected regions at a distance (Gillebert et al., 2011). Such lesions provide a spatial resolution far beyond what can be obtained from ischemic lesions of major branches of the middle cerebral artery. In order to properly evaluate the functional effects at a distance, resting-state (Carter et al., 2010; Gillebert et al., 2011; Gratton et al., 2012) or task-related fMRI (He et al., 2007; Gillebert et al., 2011) in the same patients is often essential. The value of such a multimodal imaging approach is also clear from a second case (case HH) with a focal lesion of the descending segment of left IPS, (Figure 2C), giving rise to a strictly lateralized contralesional spatial-attentional deficit (Figure 2D) (Gillebert et al., 2011). The lesion was significantly smaller than NV's lesion and confined to posterior IPS only (Figure 3). The lesion hit an area largely corresponding to IPS0/1. IPS0/1 is a visually responsive retinotopically organized region (Silver and Kastner, 2009; Bressler and Silver, 2010) that shows increased activity when attention is directed to the contralateral hemispace (Yantis et al., 2002; Vandenberghe et al., 2005; Vandenberghe and Gillebert, 2009). Apart from effects of the direction of attention, IPS0/1 is also influenced by spatiotopic mnemonic factors (Sheremata et al., 2010; Jerde and Curtis, 2013). Gillebert et al. (2011) reported the first evidence of the consequences of a lesion of IPS0/1. A lesion of left IPS0/1 preserves the visual fields leaving performance during single-target valid trials intact (Figure 2D). When the attentional demands are increased by adding an ipsilateral distracter, performance drops for contralesional targets. This is also true following an invalid spatial cue (Figure 2D). The deficit is not due to functional effects at a distance: in the IPS0/1 lesion case, both task-related and resting-state fMRI reveal that the inferior parietal lobule and the ventral attention network (Corbetta and Shulman, 2002; Corbetta et al., 2008) are functioning within a normal range (Gillebert et al., 2011). Although no right-sided isolated IPS0/1 lesions have been reported as yet, a recent study showed that repetitive transcranial magnetic stimulation (TMS) over the right IPS0/1 of healthy volunteers impairs target discrimination in the contralateral side of space (Capotosto et al., 2013). Together with results from fMRI activity in the intact brain (Yantis et al., 2002; Vandenberghe et al., 2005; Xu and Chun, 2006; Molenberghs et al., 2008; Xu and Chun, 2009), these findings can be integrated in a functional-anatomical model where IPS is subdivided in different areas (Figure 4). Posterior and middle IPS may intervene at different stages of attentional selection. The effects of IPS0/1 lesions can be accounted for by a strictly lateralized loss of attentional enhancement of a visual response to contralateral stimulation under attentionally demanding conditions (Figure 4). The middle IPS segment, on the other hand, may be involved in calibration of attentional weights for individual visuoperceptual units. According to this hypothesis, the role of IPS0/1 in attentional enhancement is defined in purely spatial coordinates while the contribution of middle IPS occurs at a stage where individual objects that occupy specific locations have already been identified (Xu and Chun, 2006, 2009; Gillebert et al., 2012b). This hypothesis is principally founded on fMRI studies in the intact brain (Vandenberghe et al., 2005; Xu and Chun, 2006, 2009) (for review, see Vandenberghe and Gillebert, 2009) and still requires further validation. It is compatible with the performance deficits seen in the two single cases with IPS lesions. By themselves, the differences in behavioral deficits between NV and HH should not be overinterpreted: the attentional deficits in these two cases do not constitute a double dissociation, the lesions do not only differ in hemispheric side but also in extent and degree of involvement of the superior parietal lobule, and the posterior portion of NV's lesion overlaps substantially with HH lesion (Figure 3). The different degree of laterality of the spatial-attentional deficit between the two cases does not necessarily mean that the right IPS has a more bilateral representation of space than the left (Weintraub and Mesulam, 1987). It can also be explained by the extension of NV's lesion into the medial wall of the superior parietal lobule which has involved in spatial shifting regardless of hemispace or direction (Vandenberghe et al., 2001a; Molenberghs et al., 2007). Based on a recent single-pulse TMS experiment (Szczepanski and Kastner, 2013), we would predict that a right-sided IPS0/1 lesion would give a similarly lateralized left-sided deficit as that provoked by the left-sided lesion in HH to the right, but this remains to be proven.

**Figure 5:**
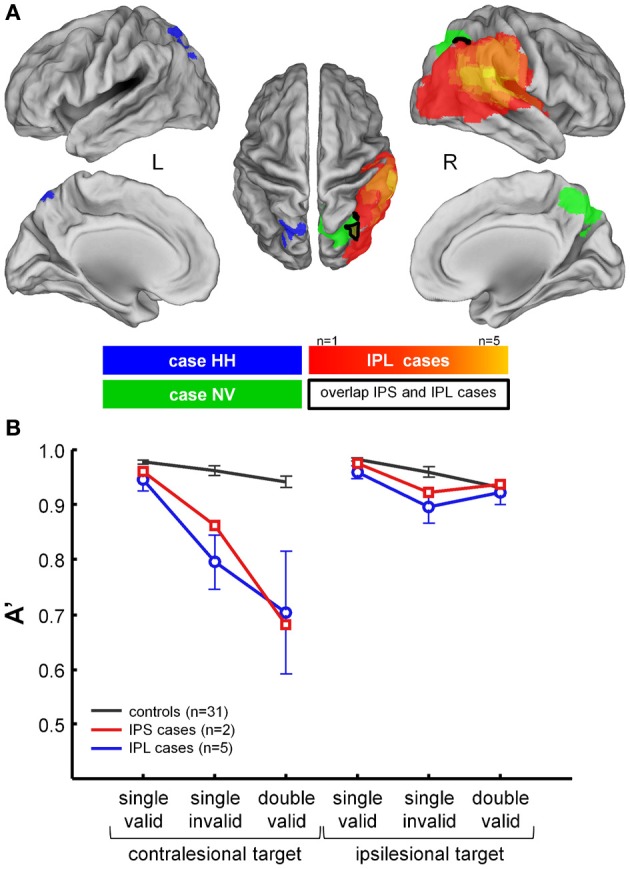
**Comparison between performance in NV and HH during the hybrid spatial cueing paradigm and five cases with classical lesions of right IPL due to ischemia of the territory of the posterior branch of the middle cerebral artery. (A)** Projection of the lesion in HH (blue), NV (green), and the IPL cases (hot scale). **(B)** Average performance in HH and NV (red), in the IPL cases (blue), and in controls (black). For further details see Gillebert et al. (2011).

Noteworthy, the behavioral deficit on invalid or competition trials following focal IPS lesions does not differ statistically from the deficits following typical inferior parietal lesions that clinically lead to hemispatial neglect or visual extinction (Figure 5) (Gillebert et al., 2011). At first this may seem to contradict canonical findings reported by Friedrich et al. (1998) but detailed analysis of the data reported by Friedrich et al. (1998) shows that in fact the two studies are compatible. The Friedrich et al. (1998) study is often interpreted as if it localizes the pathological increase of the invalidity effect to the inferior parietal cortex as opposed to superior parietal cortex. In the Friedrich et al. (1998) study, however, the main analysis did not contrast superior with inferior parietal lesions but parietal lesions extending into the superior temporal gyrus (STG) (the “TPJ” group) with parietal lesions that do not extend into STG (the “PAR” group). The “PAR” cases also had inferior parietal damage and some of the “TPJ” lesions extended into superior parietal cortex. Furthermore, the sensitivity for detecting a shifting deficit in the “PAR” group was probably relatively low given the complex factorial design (4 factors) (for a detailed discussion, see Vandenberghe et al., 2012). A voxel-based lesion-symptom mapping study in 20 left-sided neglect patients also confirmed that IPS is one of the critical regions associated with a contralateral orienting deficit and a pathological increase of the invalidity effect for contralateral targets, along with the temporoparietal junction (TPJ) and middle frontal gyrus (Ptak and Schnider, 2011).

It is important to note that the hybrid spatial cueing paradigm isolates specific components of the spatial-attentional deficits that can be seen clinically following parietal lesion. While a deficit in the competition trials compared to the single-grating trials can occur even when the clinical extinction test is within the normal range, as of yet we have not encountered clinical extinction without a deficit in the competition trials (Molenberghs et al., 2008; Gillebert et al., 2011). Likewise, patients with a deficit on target cancelation whom we tested always had a deficit on the invalid trials compared to the single-grating trials (Molenberghs et al., 2008). It is worth noting that our data are principally based on patients with unifocal cortical lesions who can do computerized testing in the acute stage with a proper sitting balance and therefore has not included patients with moderate or severe neglect.

A further parietal structure, the superior parietal lobule (SPL), has been implicated by numerous functional imaging studies (Vandenberghe et al., 2001a; Yantis et al., 2002; Shomstein and Yantis, 2004; Molenberghs et al., 2007; Serences and Yantis, 2007; Kelley et al., 2008) in the spatial displacement of the focus of attention. This is surprising as previous lesion evidence in humans provided relatively few hints for a role of superior parietal lobule in spatial shifting. According to recent evidence, however, based on the hybrid spatial cueing paradigm (Vandenberghe et al., 2012), a bilateral lesion of SPL leads to an impairment in shifting attention from the invalidly cued location to the target, regardless of its location and with preserved performance during competition trials. Medial parietal and superior parietal lesions also lead to an increased movement time during visual search (Müller-Plath et al., 2010). Recent electrophysiological recording (Brignani et al., 2009) and electrophysiological stimulation studies (Capotosto et al., 2013) have provided further evidence for the critical role of SPL in spatial shifting. A spatial shift against a sustained attention baseline provokes an event-related potential starting around 330 ms with posterior parietal distribution which does not depend on the direction of the shift, leftward or rightward (Brignani et al., 2009). Furthermore, 150 ms of repetitive TMS at 20 Hz targeting right superior parietal medial cortex 500 ms prior to onset of the shifting cue impairs target discrimination regardless of target location, in left or right visual fields (Capotosto et al., 2013).

At the moment, what is missing is statistical evidence for a double dissociation between spatial attentional processes when patients with focal lesions of parietal cortex in different locations are directly compared to each other, hence the importance of defining functional dissociations in the intact brain. The latter studies can then serve as a basis for designing paradigms in patients that may be successful in detecting double functional dissociation following lesions.

### 2.2. Hybrid spatial cueing within a cytoarchitectonic reference frame

**Figure 6:**
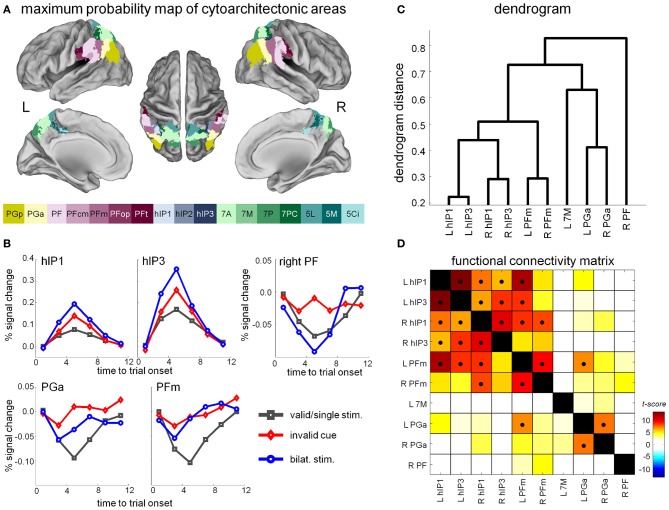
**(A)** Maximum probability maps of parietal cytoarchitectonic areas as derived from Caspers et al. (2006). **(B)** Time-activity curves in a selection of cytoarchitectonic areas that showed differential effects between the competition trials, the invalid and the valid single-target trials. **(C)** Hierarchical clustering analysis based on the time courses during resting-state fMRI in the different cytoarchitectonic areas that showed a significant competition or invalidity effect in the hybrid spatial cueing paradigm. **(D)** Functional connectivity matrix. The cross-correlation matrix is sorted on the basis of the hierarchical clustering results, so that adjacent VOIs have the most similar connectivity profiles. Significant correlations (*P* < 0.05, Bonferroni-corrected for the number of pairwise comparisons) are indicated by a black dot.

TPJ is consistently activated when reorienting attention or during breaches of expectancy (Corbetta et al., 2000). Coordinates of TPJ foci, however, vary widely between studies (Decety and Lamm, 2007; Mars et al., 2012). The inferior parietal lobule, which encompasses TPJ, is by no means homogeneous cytoarchitectonically (Caspers et al., 2006, 2011). According to cytoarchitectonic studies of postmortem brains, the angular gyrus can be subdivided into areas PGa and PGp, and the supramarginal gyrus into areas PFop, PFt, PF, PFm, and PFcm (Caspers et al., 2006, 2011) (Figure 6A). Are these human cytoarchitectonic areas differentially involved in competition vs. invalid trials (Gillebert et al., 2013) when controlling for expectancy (trial frequency kept at 20% of all trials for each of the two types of trials)? To answer this question, we applied a dual approach: starting from the cytoarchitectonical divisions, we defined volumes of interest and compared the aggregate response amplitude between the single target trials, the competition trials and the invalid trials in the hybrid spatial cueing paradigm. In a second approach, starting from the functional activity map, we overlaid the activations on the cytoarchitectonic map in order to evaluate to which degree functional boundaries coincide with cytoarchitectonic boundaries (Gillebert et al., 2013). The main challenge is the inter-individual variability in the extent and boundaries of the cytoarchitectonic areas and the probabilistic nature of assignment of voxels to a specific cytoarchitectonic area. This variability has been estimated from a relatively small set of postmortem brains (*n* = 10). This information is incorporated in the probabilistic maps (Eickhoff et al., 2005, 2006). The variability between subjects in size and borders can be relatively high in specific areas, such as hIP1-3 (Scheperjans et al., 2008).

Right PF is the only area exclusively activated for invalid vs. valid trials and not during competition trials (Gillebert et al., 2013) (Figure 6B). In right PF the difference in response amplitude between invalid and valid cueing is significantly larger than the difference between competition trials and single-target trials (Gillebert et al., 2013). In contrast, cytoarchitectonic areas hIP1 and hIP3 in IPS exhibit significantly higher activity levels during competition trials compared to invalid trials (Figure 6B). Other inferior parietal areas, such as PFm, PGa, and PGp, were bilaterally involved both in competition and invalid trials, without any significant differences between the two trial types (Figure 6B). The differential activity pattern between hIP1-3 and PF provides evidence for a functional dissociation between two types of attentional processes, those related to invalidity vs. processes of selection between competing stimuli. Note that Friedrich et al. (1998) suggested the term “extinction-like” for the invalidly cued trials but according to the above evidence, selection between competing stimuli and spatial reorienting following invalid cues are anatomically dissociable processes. A probabilistic tractography study (Caspers et al., 2011) suggested that PFm and PGa corresponded to the TPJ node of the ventral attention network (Corbetta and Shulman, 2002). Instead, our fMRI findings within a cytoarchitectonic reference frame suggest that right PF is most tightly linked to the invalidity effect in the classical spatial cueing paradigm while PFm and PGa are more generally involved in invalid as well as competition trials, at least when both trial types have a low expectancy rate.

In a second step, we superimposed the activity clusters obtained by contrasting the invalid with the valid single-target trials and by contrasting the double- with the single-target trials onto the cytoarchitectonic map (Gillebert et al., 2013). Boundaries of activation did not follow boundaries between cytoarchitectonic areas. This may suggest that, for the cognitive operations fulfilled by inferior parietal cortex, there is no functional segregation within strict cytoarchitectonic boundaries. This is also apparent from the connectivity pattern of the cytoarchitectonic areas which shows gradients between areas rather than strict segregation (Figures 6C,D) (Caspers et al., 2011; Gillebert et al., 2013). There was, however, one exception: the contrast of invalid vs. valid trials yielded a right inferior parietal activity cluster that coincided relatively closely with area PF (Gillebert et al., 2013).

### 2.3. Invalidity, competition, and connectivity

Next, we evaluated to which degree the different cytoarchitectonic areas belong to different resting-state networks (Gillebert et al., 2013). First, we derived the time courses of each of the cytoarchitectonic areas and performed a hierarchical clustering analysis (Figure 6C). Right PF was the only parietal area where the time course did not cluster with any of the other parietal regions. The time courses of hIP1-3 clustered with the time courses of PFm (Gillebert et al., 2013). When we used the cytoarchitectonic areas as seeds for a resting-state connectivity analysis across the entire brain, the patterns we obtained were in line with prior evidence (Figure 6D): Right PF belonged to a network with inferior frontal gyrus and anterior insula, hIP1-3 and PFm were connected to prefrontal cortex, and PGa was part of the default mode network (Gillebert et al., 2013). The connectivity pattern of PFm and PGa/PGp may provide hints about their functional contribution. PFm has been implicated in the multiple demand network (Duncan, 2010) or “executive control network” (Seeley et al., 2007) while PGa/PGp probably corresponds to the inferior parietal nodes of the default mode network.

According to probabilistic tractography measures of connectivity of inferior parietal cytoarchitectonic areas, the main connections of PF are with inferior frontal gyrus, insula, and cortex surrounding the central sulcus and anterior superior parietal cortex (Caspers et al., 2011). This connection may correspond to the third branch of the superior longitudinal fascicle (Thiebaut de Schotten et al., 2011). PGp, on the most posterior end, is mainly connected with occipital and temporal cortex, as well as prefrontal cortex (Caspers et al., 2011). The connection from PGp to anterior temporal cortex may correspond to the inferior longitudinal fascicle (Schmahmann and Pandya, 2006).

How do different parietal regions interact with each other and the occipital cortex to construct the attentional priority map as an emergent property? Two recent studies (Gillebert et al., 2012a; Vossel et al., 2012) addressed this issue empirically by means of Dynamic Causal Modeling (Friston et al., 2003; Penny et al., 2004). Using the spatial cueing with single-target valid trials and competition trials, Gillebert et al. (2012a) evaluated how the addition of an irrelevant distracter within-hemifield alters effective connectivity between early visual extrastriate cortex and the middle segment of IPS. When a distracter is present, the feedback connection from middle IPS to extrastriate cortex is strengthened while no effects are seen on the feedforward connections. The strengthening of the feedback connection fits with the hypothesis that middle IPS biases the competition between stimuli in upstream visual areas (Desimone and Duncan, 1995).

Vossel et al. (2012) examined how TPJ and middle IPS differentially interact with each other and with early visual cortex during orienting and reorienting of attention in a modified spatial cueing task. They observed that the feedback connection from middle IPS to extrastriate cortex was modulated by the direction of attention (leftward or rightward). Interhemispheric connections were modulated between FEF bilaterally rather than IPS. In addition, compared to validly cued targets, invalidly cued targets increased the effective connectivity from visual cortex to right TPJ, and from right TPJ to IPS and the inferior frontal gyrus (Vossel et al., 2012). The TPJ region studied by Vossel et al. (2012) corresponds principally to area PGa and PGp (Caspers et al., 2006).

## 3. The change detection paradigm

Spatial cueing paradigms in their original form or in various guises continue to engender novel insights into parietal function (Gillebert et al., 2011, 2012b, 2013; Vossel et al., 2012). As of yet, lesion studies based on spatial cueing, however, did not reveal a clear dissociation between parietal regions (Figure 5B), probably because the typical inferior parietal lesions extend beyond PF into areas such as PFm and PGa (Gillebert et al., 2013), and also into the lower bank of IPS (Molenberghs et al., 2008). Another classical paradigm probing the distribution of attentional weights as well as the capacity of visual short-term memory (VSTM) is the change detection paradigm (Luck and Vogel, 1997). It has been applied in patients (e.g., Jeneson et al., 2012) and in healthy volunteers (e.g., Todd and Marois, 2004; Xu and Chun, 2006; Mitchell and Cusack, 2008). When performing the change detection task during fMRI, IPS activity increases with an increasing number of items encoded in VSTM, and correlates across individuals with VSTM capacity (Todd and Marois, 2004; Xu and Chun, 2006).

**Figure 7:**
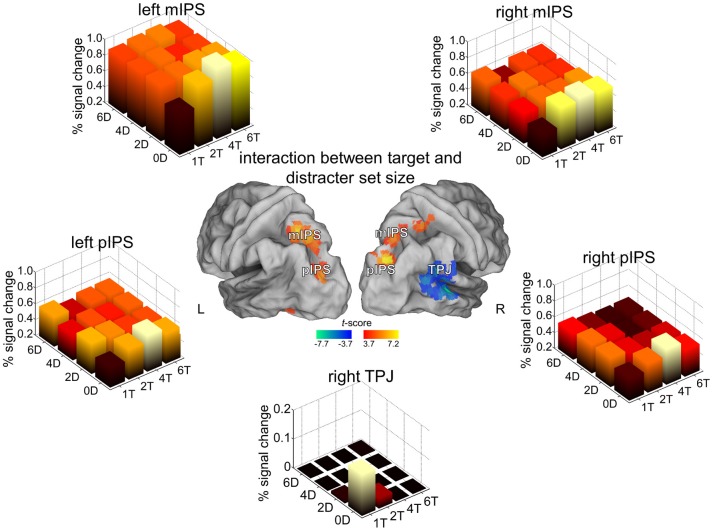
**Change detection paradigm**. For the areas where an interaction was seen between target and distracter set size, the percentage signal increase is shown for the 16 different conditions of the factorial design. For further details Gillebert et al. (2012b).

In an effort to disentangle the role of middle IPS in selection between competing stimuli (Vandenberghe et al., 2005; Molenberghs et al., 2007) from its role in visual short-term memory storage (Todd and Marois, 2004; Xu and Chun, 2006), Gillebert et al. (2012b) factorially varied the number of targets and distracters during change detection (Figures 1C,D). The behavioral relevance of the items was determined by alphanumerical class rather than by spatial location (Figure 1C). Trial-by-trial variations in the number of target and distracter items accessing VSTM were modeled mathematically based on the Theory of Visual Attention (Bundesen and Habekost, 2008; Dyrholm et al., 2011). As expected based on Todd and Marois (2004) and Xu and Chun (2006), activity in middle IPS increased asymptotically with increasing number of targets and also with increasing number of distracters (Vandenberghe et al., 2005). One of the unanticipated findings was a clear dissociation between middle IPS and right anterior IPL (encompassing mainly PF, PFm and PGa): While middle IPS increased with increasing number of targets and increasing number of distracters, anterior IPL showed highest activity when a single target was present (Figure 7). The double dissociation between IPS and right PF obtained with the change detection paradigm (Gillebert et al., 2012b) necessarily constrains the interpretations one can attribute to PF in selective attention. In the hybrid spatial cueing experiment right PF was activated by invalid trials but not during competition trials (Gillebert et al., 2013), which is in line with its exclusive activation during the single target/zero distracters condition in the change detection experiment (Gillebert et al., 2012b). We postulate that right PF functions as a target singleton detector, and is activated by conditions where a single target stands out from the background, both in sensory terms and in terms of what is expected.

Apart from this manifest dissociation, the experimental data also revealed that activity levels of middle IPS could not be reliably modeled purely on the basis of a VSTM storage account when the array contained multiple targets and distracters: There was a systematic undershoot of IPS activity under conditions of high target and high distracter set size compared to what one would predict based on the number of items entering VSTM (Figures 1D, 7) (Gillebert et al., 2012b). This may suggest that the threshold for access to VSTM can be variably adapted depending on a trade-off between easy access of targets to VSTM vs. more difficult access of distracters (Gillebert et al., 2012b).

In the change detection experiment (Gillebert et al., 2012b), posterior IPS showed a response profile that was similar to that seen in middle IPS (Figure 7). There was no laterality effect as both left and right hemispace were equally likely to contain targets and the total amount of targets and distracters was also matched across conditions between left and right hemispace. The presence of many targets and distracters elicited suppressive effects that resulted in lower response amplitudes with larger arrays, indicating that this mechanism extends to relatively early stages of the visual processing stream.

In another TVA based study (Moos et al., 2012), transcranial direct current stimulation (TDCS) was applied to the right horizontal IPS segment and its consequences were mathematically modeled based on TVA. TDCS of right middle IPS (Moos et al., 2012) led to a hemifield-independent effect on parameter α. This parameter reflects the ability to select targets and ignore distracters, and is expressed mathematically as the ratio of the attentional weight of a distractor to the attentional weight of a target at the same location. These findings are in line with a model where the middle segment of IPS plays a pivotal role in the calibration of attentional weights and the compilation of an attentional priority map (Vandenberghe et al., 2005; Vandenberghe and Gillebert, 2009) (Figure 4).

Increased activity levels during single-target conditions in the absence of distractors also occurred in PFm and PGa in the change detection experiment (Gillebert et al., 2012b). The interpretation of PFm and PGa is more tentative at the moment. PFm and PGa exhibited a relatively aspecific pattern during the hybrid spatial cueing experiment, being activated during both the invalid trials as well as the competition trials (Gillebert et al., 2013), while in the change detection experiment they were principally activated in the single target, zero distracter condition (Gillebert et al., 2012b). PGa belongs to the default-mode network (Wu et al., 2009; Uddin et al., 2010) and PFm to the multiple demand network (Duncan, 2010). Both areas have been activated by a wide variety of paradigms. The contribution of these inferior parietal areas to cognitive processes across multiple domains has been the subject of several recent reviews (e.g., Duncan, 2010; Cabeza et al., 2012; Seghier, 2013).

## 4. Conclusion

Converging evidence from functional imaging of the intact brain and parietal lesion cases indicates that middle IPS (corresponding to cytoarchitectonic areas hIPS1-3) has a critical role in selection between competing stimuli (Vandenberghe et al., 2005; Molenberghs et al., 2008; Vandenberghe and Gillebert, 2009; Ptak, 2012), superior parietal lobule in spatial-attentional shift regardless of target location (Vandenberghe et al., 2001a; Yantis et al., 2002; Molenberghs et al., 2007), and right area PF in processes related to invalidity Corbetta et al. (2000); Gillebert et al. (2013). Right PF may be particularly important when single targets stand out from the background, by virtue of a striking difference from the rest of the visual scene.

### Conflict of interest statement

The authors declare that the research was conducted in the absence of any commercial or financial relationships that could be construed as a potential conflict of interest.
